# Rapid Diagnostic Tests as a Source of Dengue Virus RNA for Envelope Gene Amplification: A Proof of Concept

**DOI:** 10.4269/ajtmh.18-0831

**Published:** 2019-06-24

**Authors:** Sarah Cassidy-Seyoum, Manivanh Vongsouvath, Onanong Sengvilaipaseuth, Malee Seephonelee, Tehmina Bharucha, Xavier de Lamballerie, Paul N. Newton, Audrey Dubot-Pérès

**Affiliations:** 1 Lao-Oxford-Mahosot Hospital-Wellcome Trust Research Unit (LOMWRU), Microbiology Laboratory, Mahosot Hospital, Vientiane, Lao P.D.R;; 2 London School of Hygiene and Tropical Medicine, London, United Kingdom;; 3 Division of Infection and Immunity, University College London, London, United Kingdom;; 4 Unité des Virus Émergents (UVE: Aix-Marseille Univ – IRD 190 – Inserm 1207 – IHU Méditerranée Infection), Marseille, France;; 5 Centre for Tropical Medicine and Global Health, Nuffield Department of Clinical Medicine, University of Oxford, Churchill Hospital, Oxford, United Kingdom

## Abstract

Molecular epidemiological data are key for dengue outbreak characterization and preparedness. However, sparse *Dengue virus* (DENV) molecular information is available in Laos because of limited resources. In this proof-of-concept study, we evaluated whether DENV1 RNA extracted from rapid diagnostic tests (RDTs) could be amplified and sequenced. The protocol for envelope gene amplification from RNA purified from RDTs was first assessed using viral isolate dilutions then conducted using 14 dengue patient sera. Envelope gene amplification was successful from patient sera with high virus titer, as was sequencing but with lower efficiency. Hence, based on our results, RDTs can be a source of DENV1 RNA for subsequent envelope gene amplification and sequencing. This is a promising tool for collecting molecular epidemiology data from rural dengue-endemic areas. However, further investigations are needed to improve assay efficiency and to assess this tool’s level of efficacy on a larger scale in the field.

In more than 100 endemic countries, there are an estimated 390 million *Dengue virus* (DENV) infections a year, of which 96 million are symptomatic.^1,2^ DENV (enveloped, single-stranded RNA) infection mostly results in a subclinical or mild febrile illness, but may occasionally cause severe dengue and lead to death. In Laos, where 3.9 million people are at risk of infection, dengue is endemic, is epidemic during the rainy season, and affects urban and rural populations.^3–6^

There are four antigenically related serotypes—DENV1–4. There is evidence to suggest that DENV serotypic and genotypic factors, such as virulence of specific serotypes and strains and the introduction of a new serotype or genotype, play roles in patient outcomes, emergence of outbreaks, and their severity.^7–10^ Therefore, molecular data can be used for epidemic modeling and outbreak preparedness. However, limited data on dengue molecular epidemiology in Laos are available, although it is known that all four DENV serotypes (DENV1–4) circulate.^4–6,11–14^

This lack of molecular epidemiological data is a result of the limited number of institutions in Laos with molecular laboratory facilities. In addition, these specialized laboratories are all located in the capital, Vientiane, and there are limited cold chain facilities to transport samples from rural areas.^15^ In those areas, the use of immunochromatographic rapid diagnostic tests (RDTs) for dengue diagnosis has recently expanded. Therefore, as a potential solution, Vongsouvath and others showed that RDTs, which are transportable at room temperature, can be used as a source for DENV RNA for small-fragment amplification using real-time reverse transcription polymerase chain reaction (RT-qPCR).^15–17^

Hence, using the Vongsouvath et al. study as a foundation, this proof-of-concept study aimed to determine whether DENV RNA of good enough quality and quantity can be extracted from RDTs to amplify and then sequence the full DENV envelope gene (Env). Access to the Env sequence would allow DENV strain characterization by phylogenetic analysis.^10,18^

We first evaluated RT-PCR assays for large-fragment amplification of RNA extracted from RDTs using dilutions of a DENV1 isolate. Second, we tested the procedure on patient samples. This tool was assessed on DENV1—the serotype that has historically caused the largest dengue burden in Laos.

The DENV1 isolate used in this study was previously obtained by virus isolation on cell culture.^4^ Replicate aliquots of 10-fold serial dilutions were performed using minimum essential medium (Gibco, ThermoFisher, Waltham, MA) until the limit of detection as determined by RT-qPCR (10^−1^ to 10^−6^).

Patient samples collected between July and October 2010 as part of a dengue molecular epidemiology study at Mahosot Hospital in Vientiane were used for this proof-of-concept study.^5^ Written informed consent for use of patient samples was obtained from all recruited patients or responsible guardians. Ethics approval was obtained from the Lao National Ethics Committee for Health Research and the Oxford Tropical Research Ethics Committee. Serum aliquots stored at −80°C from 14 patients were selected based on availability and initial RT-qPCR results; six lower titer samples and eight higher titer samples were selected.

Aliquots of each viral dilution and patient sample were loaded onto RDTs for subsequent RNA extraction. As previously described, 100 μL of the viral dilutions or patient samples was loaded on the NS1 side of the Standard Diagnostics BIOLINE Dengue Duo RDT (Standard Diagnostics, Gyeonggi-do, Republic of Korea).^15^ Subsequently, the completely dry sample pad was cut out and submitted for RNA purification using QIAamp Viral RNA Mini Kit (Qiagen, Hilden, Germany) according to previously published procedure.^15^ As reference, 140 μL of each sample was also directly extracted using QIAamp Viral RNA Mini Kit following the manufacturer’s instructions.

A pan-dengue RT-qPCR was used for DENV RNA detection and quantification, as previously described.^19^ SuperScript III Platinum One-Step qRT-PCR Kit (ThermoFisher) was used following the manufacturer’s instruction in a final volume of 25 μL, using 0.4 μM of each primer and 0.16 μM of probe with 5 μL of RNA. Ten-fold dilutions of the RNA synthetic control were used as standards for quantification (2.0 × 10 to 2.0 × 10^2^ copies/µL). Negative controls (no template) were added at each run.

For the large-fragment amplification of the full DENV1 Env, three sets of primers were designed by Dubot-Pérès et al.^4^ Conventional RT-PCRs were conducted for each primer set using the Access RT-PCR system (Promega, Madison, WI) following the manufacturer’s instruction with 5 μL of RNA in a final volume of 50 μL. The determined parameters used for optimal amplification for each primer set are displayed in Table 1.

**Table 1:** Optimal conditions for amplification by RT-PCR of the three DENV1 envelope gene fragments

Condition	Primer set		
	Primer set 1	Primer set 2	Primer set 3
Primer final concentration in the RT-PCR mix	0.4 μM	0.4 μM	0.6 μM
MgSO_4_ final concentration in the RT-PCR mix	2 mM	1 mM	1 mM
Final concentration for each dNTP in the RT-PCR mix	0.2 mM	0.2 mM	0.2 mM
Annealing temperature	59.4°C	42°C	59.4°C

Preliminary experiments (data not shown) were performed to determine the optimal primer and MgSO_4_ concentrations and annealing temperature for each primer set. Optimal conditions resulting from those experiments are displayed in the table.

The thermal cycling consisted of 45°C for 45 minutes, 94°C for 2 minutes, 40 × (94°C for 30 seconds, annealing temperature for 1 minute, and 68°C for 2 minutes), and 68°C for 7 minutes.

All RT-PCR products with successful amplification (a clear band at expected size on agarose gel) were sent to Macrogen Inc. (Seoul, South Korea) for purification and Sanger sequencing for both forward and reverse directions. The obtained sequences of the three fragments were analyzed using 4Peaks software (version 1.8, Mekentosj, Lisbon, Portugal) and assembled into a consensus sequence using Mega7 software (Pennsylvania State University, State College, PA).^20^

The three RT-PCR assays for Env amplification (Env RT-PCRs) were performed on RNA directly extracted from the dilutions of the DENV1 isolate. Successful amplification was obtained for all dilutions, except the last one, 10^−6^ (Table 2). The DENV1 isolate dilutions 10^−3^, 10^−4^, and 10^−5^ were then loaded on RDTs in triplicate. Env RT-PCRs performed on the RNA extracted from those RDTs were successful only for the highest RNA concentration dilution: 10^−3^ (Table 2). For two of the three primer sets, amplification was successful for all triplicates; however, for one primer set, only two of the three triplicates were successfully amplified.

**Table 2:** Results of RT-qPCR and envelope gene amplification performed on RNA extracted directly from DENV1 viral dilutions and after loading onto RDTs

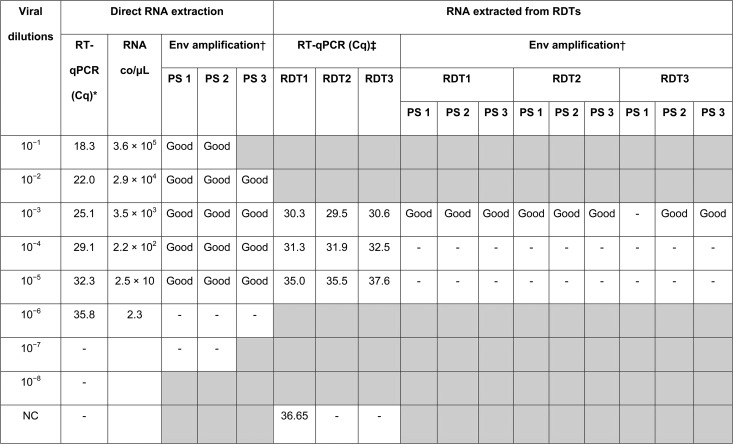

co = copies; Env = envelop gene; NC = negative control, culture medium used for dilutions; PS = primer set.

“-” stands for no Cq value or Cq ≥ 40 for RT-qPCR and for no band for conventional RT-PCR. Gray shading indicates that RT-PCR was not performed.

* Triplicate RT-qPCRs were conducted on the direct RNA extract, and the mean Cq values are displayed.

† For direct RNA extractions, 10 μL of the PCR product and 10 μL of the ladder were loaded on 1% agarose gel for primer set 1, 2, and 3 Env RT-PCRs. For RDT extractions, 20 μL of the PCR product and 20 μL of the ladder were loaded on 1% agarose gel. A “good” band is one whose intensity was comparable to or stronger than the intensity of the 1,000-bp band of HyperLadder II (corresponding to 20 ng/μL) loaded on the same gel. A band was classified as “weak” when the band was of faint intensity.

‡ Duplicate RT-qPCRs were conducted on the RNA extracted from RDTs, and the mean Cq values are displayed.

Based on the preceding results, it was expected that only patient samples with a substantial virus titer could be successfully amplified after RDT extraction. Therefore, to test this hypothesis, samples of varying virus titers were selected according to their originally obtained Cq values. Env amplification was successful for the RDT extract for three patients with the highest viral load (> 4.1 × 10^3^ copies/µL corresponding to 2.3 × 10^6^ copies/mL of blood) (Table 3). Sanger sequencing of the corresponding PCR products resulted in full (1,485 bp) and partial (983 bp) Env sequences for the two patients for whom good amplifications were obtained (Table 3, P1 and P14).

**Table 3:** Results of RT-qPCR, envelope gene amplification, and sequencing performed on RNA extracted directly from patient samples or after loading onto RDTs

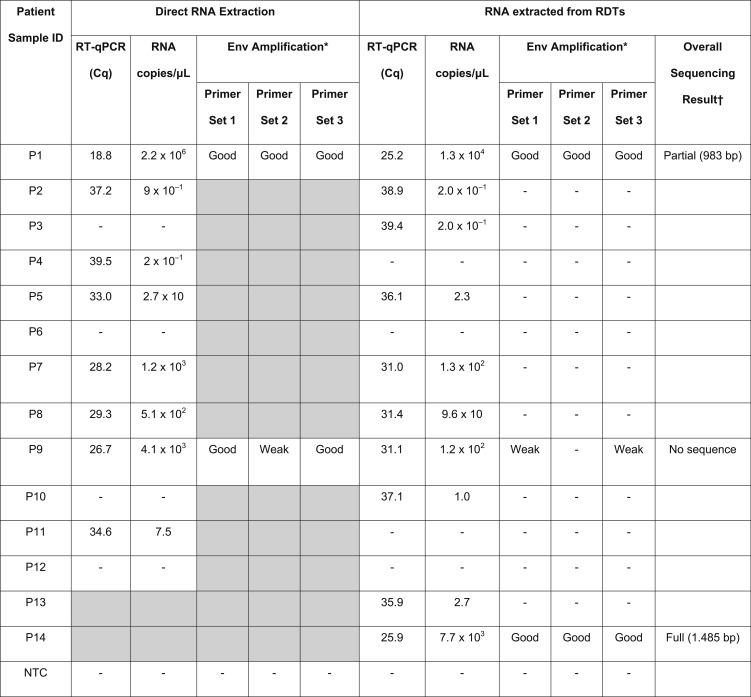

Env = envelop gene; NTC = no template control.

“-” stands for no Cq value or Cq ≥ 40 for RT-qPCR and for no band for conventional RT-PCR. “Full” stands for the full envelope gene sequence from all three primer sets and “partial” stands for a partial envelope sequence. Gray shading indicates that RT-PCR was not performed.

* Five microliter of the PCR product and 5 μL of the ladder were loaded on agarose gel for primer set 1, 2, and 3 Env RT-PCRs. A “good” band is one whose intensity was comparable to or stronger than the intensity of the 1,000-bp band of HyperLadder II (corresponding to 20 ng/μL) loaded on the same gel. A band was classified as “weak” when the band was of faint intensity.

† Sequences reported are only those of high-quality chromatograms. Reported base pair (bp) numbers are the length of the consensus sequences from the assembly of sequences from both forward and reverse directions for the three sets of primers.

For the first time, a DENV Env sequence was obtained from RDTs experimentally loaded with dengue patient sera. Using RDTs as a source of RNA for phylogenetic analyses would remove the cold chain limitation of using whole samples. Given the expanded use of dengue RDTs among Lao communities, this tool would enable access to DENV molecular information from previously inaccessible areas.

In an epidemic context, this method in its current form could be used to characterize the strain causing an outbreak through Env sequencing from patients with the highest viremia. In an outbreak, most patients are infected with the same strain. Phylogenetic analyses based on Env sequences will provide evidence regarding the origin of the DENV strain. Enhanced DENV molecular surveillance even of just high viremia patients would allow for tracking of strains and genotypes circulating countrywide over time and space. Such surveillance may enable the identification of the introduction of new strains and to better understand the dynamics of DENV strain circulation within the country and across borders. Those molecular data could be used in dengue epidemic modeling to help predict outbreaks, allowing for enhanced prevention and preparedness.

There are several factors and limitations to consider when discussing our findings and the viability and feasibility of this tool. Further optimization of the conventional RT-PCR assay and sequencing are likely to permit successful amplification for patients with lower viral loads. First, a simple adjustment would be to increase the volume of the RNA template used for Env RT-PCRs. Second, long-range PCR assays may be more efficient at amplifying the RDT-extracted RNA. Finally, cloning of amplicons or using high-throughput sequencing may improve sequencing results.

Other factors limiting the efficiency of the technique include a potential loss of RNA when extracting from RDTs versus direct extraction, and the storage duration of the samples used.^15^ The samples were collected 7 years before this study and stored at −80°C; therefore, RNA degradation over time is likely to have affected amplification and sequencing results. Hence, testing the procedure with prospectively collected patient samples is required to determine if better results can be obtained.

Ideally, our protocol should be tested on numerous RDTs from the field that have been stored and transported at room temperature to ascertain whether the use of RDTs as a source of RNA for amplification is practically possible on a large scale. If temperature were to be a limiting factor, additional techniques, such as adding RNAlater to the RDTs after being read, could mitigate potential RNA degradation. In addition, the protocols need to be tested on the remaining three serotypes and on other RDT brands that may be used across the country.

If the RT-PCR assay sensitivity were increased and the approach were more thoroughly tested on RDTs from the field, using RDTs for sample collection would enable access to more dengue epidemiological data, producing a more complete picture of the complex DENV strain dynamics in Laos.^5^ This would likely improve the accuracy of outbreak forecasting at national and regional levels. Therefore, this proof-of-concept study showed that the use of RDTs as a source of RNA for DENV molecular epidemiology analyses is a promising tool that merits further investigation.
